# Mapping Spatiotemporal Metabolic Perturbations in Alloxan-Induced Diabetic Rat Kidneys Using Spatial Metabolomics and Proteomic Integration

**DOI:** 10.3390/metabo16060355

**Published:** 2026-05-25

**Authors:** Tianfang Lan, Caiying Liu, Xingyu Zhang, Xiaoyu Zhang, Yuchen Liu, Wenxuan Shao, Zhonghua Wang

**Affiliations:** 1 Center for Imaging and Systems Biology, College of Life and Environmental Sciences, Minzu University of China, Beijing 100081, China; 23302551@muc.edu.cn (T.L.); 24011671@muc.edu.cn (C.L.); 24302635@muc.edu.cn (X.Z.); 24302654@muc.edu.cn (X.Z.); 24011748@muc.edu.cn (Y.L.); 24011864@muc.edu.cn (W.S.); 2 Key Laboratory of Mass Spectrometry Imaging and Metabolomics, Minzu University of China, National Ethnic Affairs Commission, Beijing 100081, China

**Keywords:** spatial metabolomics, mass spectrometry imaging, diabetic nephropathy, alloxan-induced diabetes, renal metabolic heterogeneity

## Abstract

**Background:** Diabetic nephropathy (DN) is characterized by complex and region-specific metabolic dysregulation that is not captured by conventional biomarkers. However, the spatiotemporal organization of metabolic alterations across renal compartments in type 1 diabetes remains poorly understood. **Methods:** In this study, spatial metabolomics based on air flow-assisted desorption electrospray ionization mass spectrometry imaging (AFADESI-MSI) was applied to investigate metabolic alterations in kidney tissues from alloxan-induced diabetic rats at 4 and 8 weeks post-induction. Complementary LC–MS/MS metabolite profiling and label-free proteomic analysis were performed to support pathway interpretation. **Results:** Spatial metabolomics revealed pronounced region- and time-dependent metabolic reprogramming in diabetic kidneys. Early-stage (DN-4w) changes were characterized by elevated glucose and activation of glucose-associated pathways, including the polyol pathway, accompanied by accumulation of acylcarnitines and lipid intermediates, indicating metabolic substrate overload. At later stages (DN-8w), glucose and related metabolites declined, reflecting impaired metabolic capacity and mitochondrial dysfunction. Broad remodeling of lipid metabolism, including glycerophospholipids, fatty acids, and hexosylceramide, was observed, along with dysregulation of amino acid metabolism and redox-related pathways. These alterations exhibited clear regional heterogeneity across renal cortex and medulla, highlighting compartment-specific metabolic vulnerability. **Conclusions:** This study provides a comprehensive spatial characterization of metabolic perturbations during DN progression, revealing coordinated alterations in glucose utilization, lipid metabolism, and mitochondrial function. The findings demonstrate the value of spatial metabolomics in uncovering region-specific metabolic mechanisms and provide new insights into the pathogenesis of diabetic nephropathy.

## 1. Introduction

Diabetic nephropathy (DN) is one of the most prevalent and severe microvascular complications of diabetes mellitus and remains the leading cause of end-stage renal disease worldwide. It is characterized by progressive glomerular and tubular injury, mesangial expansion, extracellular matrix accumulation, and interstitial fibrosis, ultimately leading to irreversible renal dysfunction [1]. Despite the widespread clinical use of biomarkers such as albuminuria and estimated glomerular filtration rate (eGFR), these indicators provide only limited insight into the underlying molecular events and fail to capture the spatial heterogeneity of renal pathology [2]. Given the highly structured organization of the kidney, where distinct regions such as the cortex, outer medulla, and inner medulla exhibit specialized metabolic functions, elucidating region-specific and time-dependent metabolic alterations is critical for understanding DN pathogenesis and identifying novel therapeutic targets.

Animal models have been instrumental in advancing the understanding of DN pathogenesis and in evaluating therapeutic interventions. Both spontaneous and experimentally induced models have been established, each capturing distinct features of diabetic kidney injury [3,4]. In parallel, developments in mass spectrometry imaging (MSI) have enabled label-free, spatially resolved profiling of endogenous metabolites directly from tissue sections, providing powerful tools to investigate metabolic heterogeneity in situ [5]. Among these approaches, air flow-assisted desorption electrospray ionization mass spectrometry imaging (AFADESI-MSI) offers broad metabolite coverage, high sensitivity, and minimal sample preparation, making it particularly well suited for spatial metabolomics analysis of complex tissues.

In our previous work, AFADESI-MSI-based spatial metabolomics was successfully applied to characterize region-specific metabolic reprogramming in both a high-fat diet/streptozotocin (HFD/STZ)-induced type 2 diabetic rat model and a spontaneous DN model in db/db mice [5,6]. These studies revealed pronounced spatial heterogeneity in pathways related to amino acid, lipid, and energy metabolism, particularly in advanced stages of disease. However, these models predominantly reflect type 2 diabetes and insulin resistance-associated renal injury. In contrast, the spatial metabolic features associated with type 1 diabetes-induced DN remain insufficiently explored, particularly with respect to their temporal progression across renal compartments.

The alloxan-induced diabetic model is widely used to mimic type 1 diabetes, as it selectively destroys pancreatic β-cells and induces persistent hyperglycemia and systemic metabolic disturbances [7]. While the renal histopathological and functional changes in alloxan-induced DN have been previously reported, the spatial metabolic landscape and its progression within kidney regions remain unclear. This limits our understanding of how diabetes-induced metabolic dysregulation manifests across renal compartments such as the cortex, medulla, and papilla during disease development.

In this study, we applied AFADESI-MSI-based spatial metabolomics to investigate region-specific metabolic alterations in the kidneys of rats with alloxan-induced diabetes at 4 and 8 weeks post-induction. This approach enabled spatial mapping of metabolic perturbations across distinct renal compartments and facilitated the evaluation of stage-associated changes during disease progression. In addition, complementary proteomic analysis at the early stage was performed to provide molecular context for the observed metabolic alterations. By resolving compartment-specific metabolic responses to hyperglycemia, this study aims to enhance the understanding of metabolic heterogeneity in diabetic nephropathy and to demonstrate the utility of spatial metabolomics in studying diabetic kidney disease.

## 2. Materials and Methods

### 2.1. Chemicals

LC-MS-grade acetonitrile (ACN) and methanol (MeOH) were purchased from Merck (Muskegon, MI, USA). Ultrapure water was obtained from Wahaha (Hangzhou, China). Alloxan monohydrate and sodium citrate were purchased from Sigma-Aldrich (St. Louis, MO, USA).

### 2.2. Animal Models

Six-week-old male Wistar rats (175–210 g) were obtained from Vital River Laboratory Animal Technology Co., Ltd. (Beijing, China). Animals were housed under specific pathogen-free (SPF) conditions at 23 ± 3 °C with 50 ± 10% relative humidity and a 12 h light/dark cycle, with standard chow and water ad libitum. Rats were randomly assigned to three groups: control (*n* = 6), DN-4 week (*n* = 6), and DN-8 week (*n* = 6).

To induce diabetes, rats in the DN groups received a single intraperitoneal injection of freshly prepared alloxan monohydrate (200 mg/kg body weight) dissolved in sterile normal saline after an overnight fast. Control rats received an equivalent volume of sterile saline. Fasting blood glucose (FBG) was measured at 72 h and 7 days post-injection using a glucometer (OneTouch Ultra, New Brunswick, NJ, USA, Johnson & Johnson). Rats with FBG ≥ 16.7 mmol/L on two consecutive measurements were considered diabetic and included. Diabetic rats were euthanized at 4 or 8 weeks after alloxan administration (DN-4w and DN-8w, respectively), whereas control rats were euthanized at week 4. At each time point, blood was collected from the abdominal aorta under anesthesia. Kidneys were rapidly excised, rinsed with ice-cold saline, snap-frozen in liquid nitrogen, and stored at −80 °C until analysis. All procedures were approved by the Ethics Committee of Minzu University of China (No. ECMUC2023003AA; Beijing, China).

### 2.3. Histopathology

Frozen kidneys were sectioned at 10 μm thickness at −22 °C using a Leica CM1860 cryostat (Leica Microsystems, Wetzlar, Germany) and mounted on adhesion microscope slides (Thermo Scientific, Waltham, MA, USA). Hematoxylin and eosin (H&E) staining was performed to evaluate histopathological changes.

### 2.4. AFADESI–MSI Analysis

MSI was performed using an AFADESI-MSI platform consisting of a home-built AFADESI ion source coupled to a Q-OT-qIT hybrid mass spectrometer (Orbitrap Fusion Lumos; Thermo Fisher Scientific, Waltham, MA, USA). Data were acquired in positive and negative full-scan MS modes over *m*/*z* 100–1000 at a resolution of 120,000. Detailed parameters are provided in the Supporting Information (Table S1).

### 2.5. LC–MS/MS Analysis

To provide supporting MS/MS evidence for metabolite identification, kidney tissue homogenates were subjected to liquid chromatography-tandem mass spectrometry (LC-MS/MS) analysis. The system consisted of an UltiMate 3000 series HPLC system (Thermo Fisher Scientific, Waltham, MA, USA) coupled to a Q-OT-qIT hybrid mass spectrometer (Orbitrap Fusion Lumos, Thermo Fisher Scientific, Waltham, MA, USA). Analyses were conducted in both positive and negative ionization modes. The comprehensive protocols for sample preparation and the detailed instrumental parameters for LC-MS/MS are provided in the Supporting Information.

### 2.6. Proteomic Analysis

Label-free proteomic analysis was performed on kidney tissues from control and DN-4w groups. Protein extraction, digestion, and LC-MS/MS analysis were carried out by Novogene Co., Ltd. (Beijing, China) following standard protocols. Briefly, ~100 mg of tissue was ground in liquid nitrogen and lysed in SDT buffer (100 mM NaCl, 1% DTT), followed by ultrasonication, heating at 95 °C, and centrifugation. The supernatant was alkylated with iodoacetamide, precipitated with cold acetone, and re-dissolved in buffer (8 M urea, 100 mM TEAB, pH 8.5). Proteins were digested with trypsin at 37 °C (4 h followed by overnight digestion with additional trypsin and CaCl_2_). The digestion was terminated with formic acid (pH < 3), and peptides were desalted using C18 columns, eluted with 70% acetonitrile containing 0.1% formic acid, lyophilized, and reconstituted for analysis. Approximately 1 μg of peptides was analyzed by high-resolution LC–MS/MS. Protein identification and quantification were performed using DIA-NN software (version 1.8.1) against the UniProt Rattus norvegicus database (January 2024). Detailed experimental procedures, instrument parameters, and data processing workflows are provided in the Supporting Information.

### 2.7. Data Processing and Statistical Analysis

Raw data files acquired in positive and negative ion modes were converted to .cdf format using Xcalibur 4.0.2 (Thermo Scientific, Waltham, MA, USA) and imported into MassImager (MassImager 2.0, Beijing, China) for background subtraction, normalization, and ion image reconstruction. Total ion current (TIC) normalization was applied at the pixel level prior to ion image reconstruction. Regions of interest (ROIs) were defined based on histological guidance and manually delineated to ensure consistent anatomical regions across samples. ROI-level data were exported as .txt files.

The datasets were imported into MarkerView™ 1.2.1 (AB SCIEX, Toronto, ON, Canada) for peak picking and alignment. Relative intensities were calculated for each ROI using total ion current (TIC) normalization. Group comparisons were performed using Student’s *t*-test, and metabolites with *p* < 0.05 were considered discriminatory.

Venn diagrams were generated using Venny 2.1 (https://bioinfogp.cnb.csic.es/tools/venny/index.html (accessed on 20 February 2026)) by pasting up to four metabolite lists (one element per line) into the input fields; intersections were inspected by clicking the corresponding numbers in the diagram.

Pathway analysis was conducted using MetaboAnalyst 6.0 (https://www.metaboanalyst.ca (accessed on 6 February 2026)) via the Pathway Analysis module. HMDB identifiers of differentially abundant metabolites were uploaded as a compound list and the organism was set to Rattus norvegicus (rat). Enrichment and topology results were exported (including the “Match Status” table) as .csv files, and figures were generated in R.

Protein–protein interactions (PPIs) were predicted using the STRING database (https://string-db.org (accessed on 6 February 2026)) and visualized with Cytoscape (Version 3.10.4) [8]. Proteins not connected within the interaction network were excluded from further analysis. Correlation analysis between differentially expressed proteins (DEPs) and differentially expressed metabolites (DEMs) was performed using the R package corrplot (Version 0.95), after filtering for DEPs and DEMs co-enriched in KEGG pathways.

### 2.8. Metabolite Identification

Putative metabolite identification was performed according to previously described protocols [9,10]. Briefly, accurate *m*/*z* features detected by AFADESI-MSI were first matched against an in-house, custom-built database using a mass error tolerance of ±5 ppm to enable rapid annotation of known compounds. Common adduct forms were considered, including [M + H]^+^, [M + Na]^+^, and [M + K]^+^ in positive ion mode, and [M − H]^−^ and [M + Cl]^−^ in negative ion mode, along with potential in-source fragments reported in metabolomics databases. Candidate annotations were then further evaluated using LC–MS/MS analysis of kidney tissue homogenates. MS/MS spectra were interpreted based on characteristic fragmentation patterns, and identifications were considered supported when at least two diagnostic fragment ions matched reference spectra from public databases. To reduce misannotation, theoretical *m*/*z* values for plausible adducts and in-source fragments were calculated in R and used during annotation. In addition, concordant spatial localization of different adduct forms of the same metabolite observed by AFADESI-MSI was used as supportive evidence for identification.

## 3. Results

### 3.1. Assessment of Renal Injury in Alloxan-Induced Diabetic Rats

**Figure 1:**
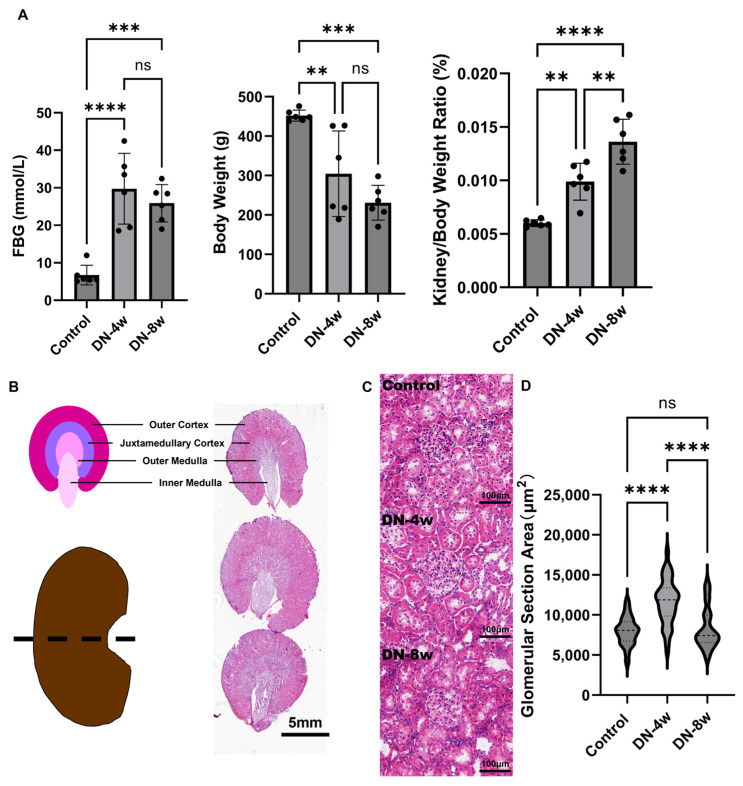
Fasting blood glucose, body weight and kidney/body weight ratio of alloxan-induced diabetic rats (**A**). Schematic diagram of rat kidney tissue segmentation and the overall H&E staining image of kidney cross sections. Scale bar = 5 mm (**B**). Detailed H&E staining images of glomerulus across the groups, scale bar = 100 μm (**C**). ANOVA of glomerular areas on sections among samples in groups, at least *n* = 200 each group (**D**). As compared to any group, ** *p* < 0.01, *** *p* < 0.001 and **** *p* < 0.0001. Control rat: control group rat; DN-4w rat: tissue collection 4 weeks after alloxan administration.

Fasting blood glucose (FBG), body weight, and kidney-to-body weight ratio are summarized in Figure 1A–D. Compared with controls, FBG levels were significantly elevated in both DN-4w and DN-8w groups and increased further over time, indicating persistent and progressive hyperglycemia. In parallel, diabetic rats exhibited a significant, time-dependent reduction in body weight (*p* < 0.01), reflecting systemic metabolic impairment. Despite weight loss, the kidney-to-body weight ratio was significantly increased in diabetic groups and further elevated at 8 weeks (*p* < 0.01), suggesting progressive renal hypertrophy during disease development.

Histological analysis revealed clear structural alterations in diabetic kidneys (Figure 1B,C), including glomerular enlargement, mesangial expansion, and increased cellularity. Quantitative analysis confirmed a significant increase in glomerular area in DN groups, which was more pronounced at 8 weeks (Figure 1D), indicating time-dependent progression of renal injury.

Overall, these physiological and histopathological changes confirm the successful establishment of the alloxan-induced diabetic nephropathy model and provide a solid foundation for subsequent spatial metabolomics analysis.

### 3.2. AFADESI–MSI Analysis of Kidneys from Alloxan-Induced DN Rats

Frozen kidney sections from control and alloxan-induced diabetic rats were analyzed by AFADESI–MSI to characterize spatially resolved metabolic alterations during DN progression. Based on H&E staining, each section was segmented into four major anatomical regions: whole kidney (W), cortex (C), outer medulla (OM), and inner medulla (IM). To further resolve cortical heterogeneity, the cortex was manually subdivided into outer cortex (OC) and juxtamedullary cortex (JC) using the spatial distribution patterns of region-enriched phospholipids described in our previous work. This yielded five ROIs in total (W, OC, JC, OM, IM) for downstream region-wise comparisons.

In positive-ion mode, 2044/1735/1750/1667/1643 ion features were detected in W/OC/JC/OM/IM of DN-4w kidneys, whereas 1841/1525/1750/1526/1615 features were detected in the corresponding regions of DN-8w kidneys. In negative-ion mode, 1308/1248/1164/1063/1235 features were detected across W/OC/JC/OM/IM in diabetic kidneys, compared with 1249/1011/1112/1011/1059 features in the corresponding regions of control kidneys.

**Figure 2:**
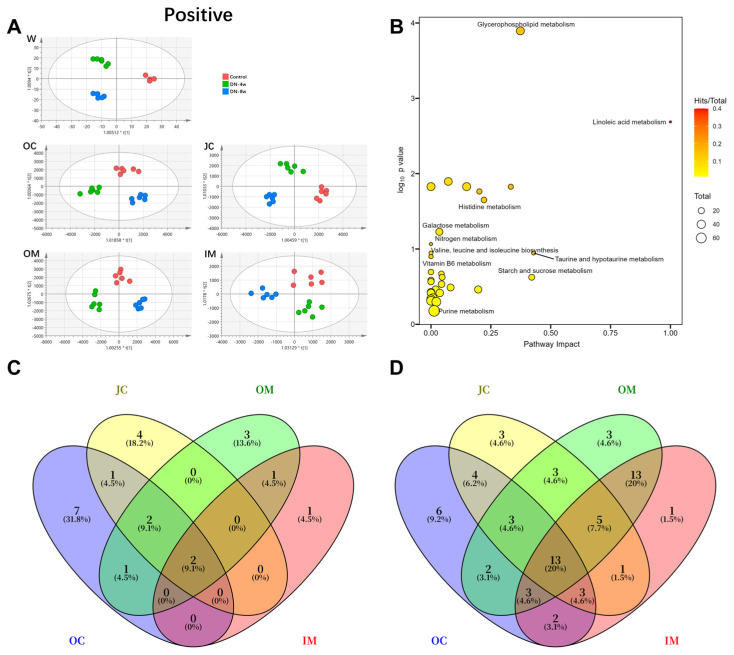
OPLS-DA score plots based on the positive AFADESI-MSI data from the control group (*n* = 6), DN-4w group (*n* = 6) and DN-8w group (*n* = 6). (**A**) Pathway enrichment analysis of differentially abundant metabolites associated with diabetic nephropathy in alloxan-induced diabetic rats (**B**) Venn diagram of significant differential ions between Con and DN-4w detected in multiregional renal regions under positive and negative ion mode respectively. (**C**,**D**) OC: outer cortex. JC: juxtamedullary cortex. OM: outer medulla. IM: inner medulla.

To elucidate the spatial metabolic characteristics during the progression of diabetic nephropathy (DN), AFADESI-MSI profiles of rat kidney sections from the control, DN-4w, and DN-8w groups were analyzed using supervised orthogonal partial least squares discriminant analysis (OPLS-DA). The OPLS-DA score plot (Figure 2A and Figure S3B) showed clear separation among the groups, particularly between DN and control samples, indicating substantial metabolic divergence associated with diabetic kidney injury. Model validation metrics, including R^2^ (explained variance) and Q^2^ (predictive ability), as well as permutation test results, are provided in the Supporting Information (Table S3).

To identify disease-associated changes within each compartment, metabolic profiles from each ROI were compared between groups using Student’s *t*-test, and ions with *p* < 0.05 were considered discriminatory. In positive-ion mode, 335/262/375/217/235 discriminatory variables were obtained in W/OC/JC/OM/IM of DN-4w rats, while 716/618/704/512/332 discriminatory variables were obtained in the corresponding regions of DN-8w rats. In negative-ion mode, 198/229/148/205/305 discriminatory variables were obtained in W/OC/JC/OM/IM of DN-4w rats, while 444/438/463/406/541 discriminatory variables were obtained in the corresponding regions of DN-8w rats. The increased number of discriminatory ions at 8 weeks indicates that metabolic perturbations became more extensive with disease progression, and the ROI-dependent differences indicate compartment-specific metabolic responses.

In total, 32 and 40 differentially abundant metabolites were confidently identified in positive- and negative-ion modes, respectively, and their region-restricted changes are summarized in Tables S4 and S5, Figure 2C,D and Figure S1. Pathway analysis (MetaboAnalyst 6.0) identified 25 impacted pathways, led by linoleic acid metabolism, taurine and hypotaurine metabolism, starch and sucrose metabolism, glycerophospholipid metabolism and Histidine metabolism (Figure 2B and Figure S3A).

### 3.3. Proteome Analysis of Kidneys from Alloxan-Induced DN Rats

In total, 9326 unique proteins were identified and quantified by mapping peptide reads to the Rattus Norvegicus protein database via DIA-NN. Protein quantification results were statistically analyzed using the *t*-test, and differentially expressed proteins (DEPs) were defined as those with significant quantitative differences between the experimental and control groups (*p* < 0.05, FC > 1.2 or FC < 0.83). A total of 297 proteins are considered differentially expressed.

**Figure 3:**
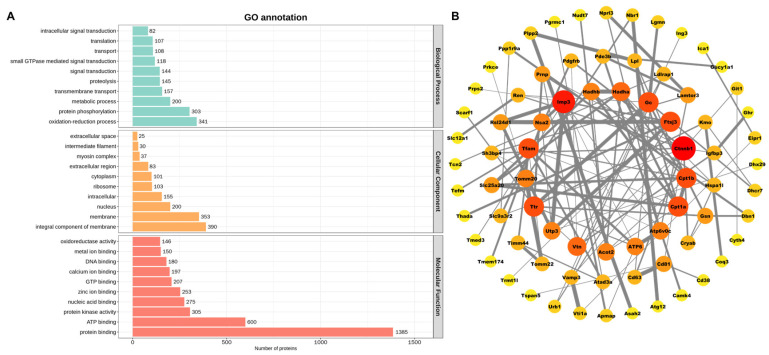
Histogram of Go functional enrichment of all 297 DEPs. (**A**) Histogram of visualized protein–protein interactions of 75 DEPs can be integrated in the largest network. The nodes are placed in circle according to hierarchy of interaction degrees (**B**).

Among all DEPs, 75 of these proteins are annotated by STRINGdb to be in a large network. The top 10 proteins in terms of the most connection nodes, as indicated in the PPI graph, are considered as hub genes (Figure 3B). Cpt1a, Cpt1b and Hadha are crucial genes of the beta-oxidation of fatty acids [11]. The upregulation of these three genes simply indicates a change in main source of energy in diabetic kidney. Imp3 and Ftsj3 are considered as regulation factors in ribosome assembling and RNA modifying respectively [12,13], which indicates a stress response of dysregulated metabolism and oxidative stress as the subsequence. Ttr and Gc have been downregulated, which are targets of thyroxine and vitamin D. Fewer receptors means higher ligand concentration is needed to achieve the same response, meanwhile a few previous articles observed that the supplementation of levothyroxine and vitamin D is associated with lower risk of diabetic nephropathy in humans [14,15]. Vtn upregulation is considered as a corresponsive factor in early kidney fibrosis [16]. The function of the hub genes shows a changed metabolism and therefore damage to the kidney.

#### Protein Correlation with Metabolites in DEPs

**Figure 4:**
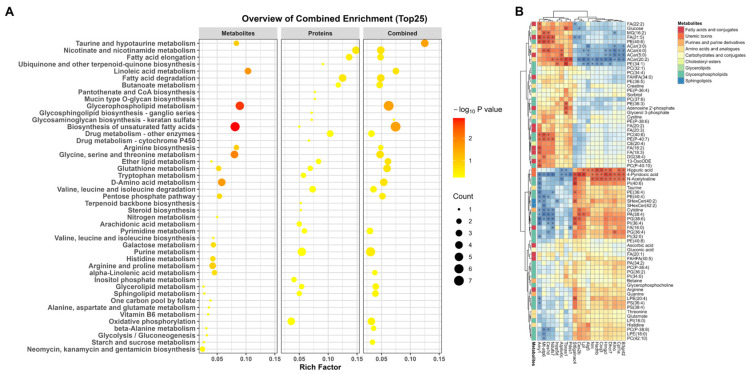
Histogram of compartment of metabolites, proteins, and a combination of metabolites and proteins enriched to KEGG Rattus Norvegicus metabolism pathways. (**A**) Histogram of Pearson correlation analysis between 20 proteins which can be mapped in KEGG Rattus Norvegicus metabolism pathway and all 72 DEMs. * *p* < 0.05 (**B**).

All 297 proteins are KEGG enriched by R package clusterProfiler. A custom kegg pathway mapping table that only contains the “Metabolism” category was created by R package massdatabase; thus, protein enrichment entries can be matched with the metabolite enrichment entries from Metaboanalyst. A protein–metabolite combined expression table was used to filter proteins that can be enriched into identical entries with DEMs (Figure 4A). The top 25 *p*-values of the entries were filtered to be represented in the histograms.

It should be noted that the enrichments of differently expressed metabolites and proteins are not entirely matched. A total of 39 proteins are able to be enriched in metabolism pathways, mainly nicotinate and nicotinamide metabolism, fatty acid elongation, fatty acid degradation, and butanoate metabolism. However, 20 proteins can be enriched with metabolites, which includes biosynthesis of unsaturated fatty acids, ether lipid metabolism and glutathione metabolism. These terms are seemingly identical to the major metabolite categories involved in DEMs.

To see if there was a more clear relation between these 20 proteins and 72 metabolites, a Pearson correlation analysis was conducted to figure out the co-expression relationship (Figure 4B). We note that these 20 proteins are gathered into two clusters. These two clusters show an impairment between metabolites and proteins rather than only the relative expression in the proteome. Cluster 1, including B3gnt2, Cpt1a, Kmo, Dhcr7 and Hmgcl, etc., is negatively correlated with acetyl carnitines like ACar(4:0), and positively correlated with uremic toxins such as hippuric acid, 4-pyridoxic acid and N-acetylvaline. Cluster 2, including Ndufs7, Ces1d, and Mt-atp6, are negatively correlated with 4-pyridoxic acid, cytidine, and a few phosphoglycerolipids, and positively correlated with acetyl carnitines. An impairment of acetyl carnitine accumulation and oxidative phosphorylation is clear. Pdss1 and Coq3 disturbance indicates a struct in the coenzyme Q synthesis pathway [17]. A relative overexpression of Ndufs7 and Mt-atp6 to acetyl carnitines indicates an expanded scale of oxidative phosphorylation [18,19]; however, the impairment of Cpt1a, Hadhb, Lpl and Ces2b restricted the transportation and oxidation in free fatty acids, causing it accumulate in the diabetic kidney [20,21,22,23]. These impairments eventually cause a negatively corresponded relationship of Hmgcl and ACar, which is identical to an obstructed fatty acid oxidation changing to ketogenesis [24]. The impairment of Dhcr7 makes it unable to repair mitochondrial membrane damage [25], and Nnt impairment makes it unable to remove oxidative species via generating NADPH [26]. The overall protein expression pattern is highly related to mitochondrial energy metabolism, lipid metabolic homeostasis, and the efficiency of mitochondrial oxidative damage control in the kidney, suggesting that they may be involved in key processes that maintain or disrupt renal metabolic balance.

**Figure 5:**
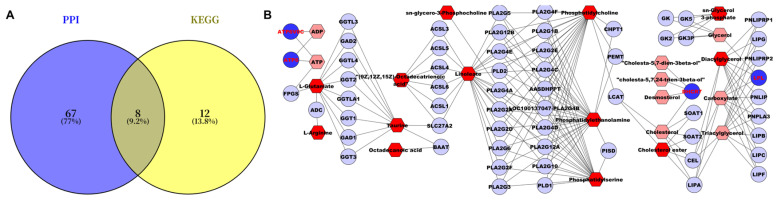
Venn diagram of intersection in two set of DEP analysis. (**A**) Histogram of connnections between 8 crucial enzymes and 12 crucial metabolites found to be differently expressed (**B**).

The intersection of proteins important in the PPI network that enrich with metabolites are shown to be Atp6v0c, Coq3, Cpt1a, Dhcr7, Hadhb, Kmo, Lpl and Mt-atp6 (Figure 5A). Metascape shows that four proteins are in the enzyme–metabolite network. These eight proteins and 12 integrated metabolites could be characteristic phenotypes of T1DM nephropathy. The nodes in between the highlighted nodes need further study to reveal greater connections between proteomes and metabolomes (Figure 5B).

## 4. Discussion

### 4.1. Amino Acid and Nitrogen Metabolism

**Figure 6:**
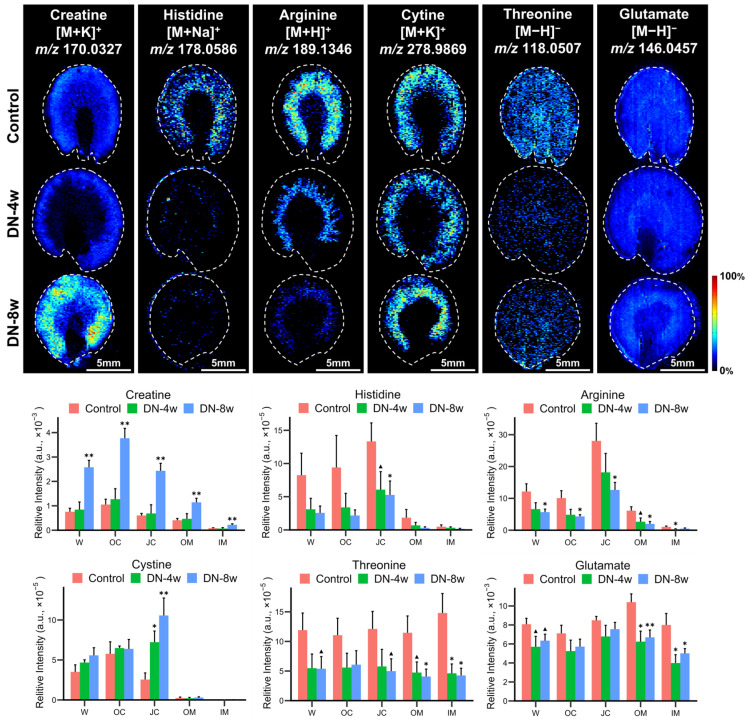
Spatial distribution of differentially abundant amino acids in alloxan-induced diabetic rats, with histogram of representative metabolites’ total ion current intensity per microdomain involved in amino acid and nitrogen metabolism detected through the AFADESI-MSI analysis in the kidneys of the groups. OC: outer cortex. JC: juxtamedullary cortex. OM: outer medulla. IM: inner medulla. As compared to control group, ▴ *p* < 0.1, * *p* < 0.05 and ** *p* < 0.01 (*n* = 6; mean ± SEM).

AFADESI–MSI revealed significant changes in several amino acids and related metabolites, including creatine, histidine, arginine, threonine, cystine, and glutamate (Figure 6). As these metabolites are tightly coupled to nitrogen handling, redox buffering, and inflammatory signaling, their coordinated dysregulation suggests that diabetic kidneys undergo substantial metabolic stress in a region- and time-dependent manner. Among them, glutamate is a key precursor for glutathione biosynthesis and therefore contributes directly to intracellular redox homeostasis [27]. Perturbation of glutamate-associated pathways is consistent with enhanced oxidative stress, a recognized driver of DN initiation and progression. Arginine, the substrate for nitric oxide synthase, links amino acid metabolism to vascular function; impaired arginine availability can reduce nitric oxide (NO) production, promoting endothelial dysfunction and aggravating glomerular injury [28]. In addition, altered cystine levels and increased proline-containing dipeptides may reflect disturbed protein turnover and activation of pro-fibrotic programs, consistent with extracellular matrix accumulation and renal fibrosis [29]. Threonine also connects to one-carbon metabolism and glycine-related pathways, which support methyl-group transfer reactions and antioxidant capacity, implying that shifts in threonine metabolism may contribute to both epigenetic regulation and redox imbalance in diabetic kidneys [30].

### 4.2. Energy Metabolism and the Polyol Pathway

**Figure 7:**
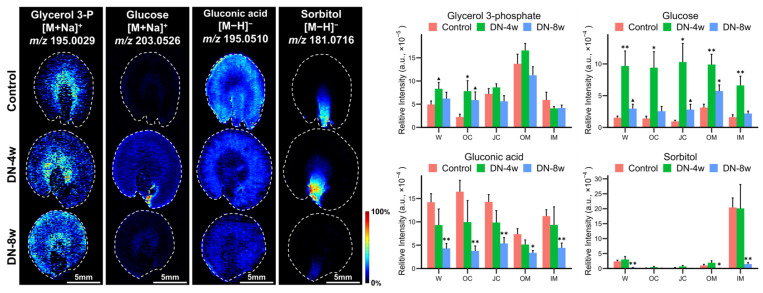
Spatial distribution of differentially abundant energy metabolism related compounds in alloxan-induced diabetic rats with histogram of representative metabolites’ total ion current intensity per microdomain involved in energy metabolism and the polyol pathway detected through the AFADESI-MSI analysis in the kidneys of the groups. OC: outer cortex. JC: juxtamedullary cortex. OM: outer medulla. IM: inner medulla. As compared to control group, ▴ *p* < 0.1, * *p* < 0.05 and ** *p* < 0.01 (*n* = 6; mean ± SEM).

Marked changes in glucose, sorbitol, gluconic acid, and glycerol-3-phosphate (Figure 7) indicate substantial disruption of renal energy metabolism under sustained hyperglycemia and suggest increased polyol pathway activity. In this pathway, aldose reductase converts glucose to sorbitol, a reaction that consumes NADPH. Enhanced flux therefore depletes reducing equivalents needed for antioxidant systems (e.g., glutathione recycling), thereby amplifying oxidative stress. In addition, sorbitol accumulation within renal tissue can promote osmotic stress, which has been linked to tubular injury in diabetes [31].

Concurrently, altered levels of intermediates related to glycolytic input and lipid–carbohydrate shuttling (e.g., glycerol-3-phosphate) are consistent with disturbed substrate utilization and may reflect mitochondrial dysfunction and impaired oxidative phosphorylation. Such bioenergetic impairment is a recognized contributor to tubular vulnerability, facilitating stress responses, apoptosis, and ultimately progressive renal dysfunction during DN.

### 4.3. Acylcarnitines and Fatty Acid β-Oxidation

**Figure 8:**
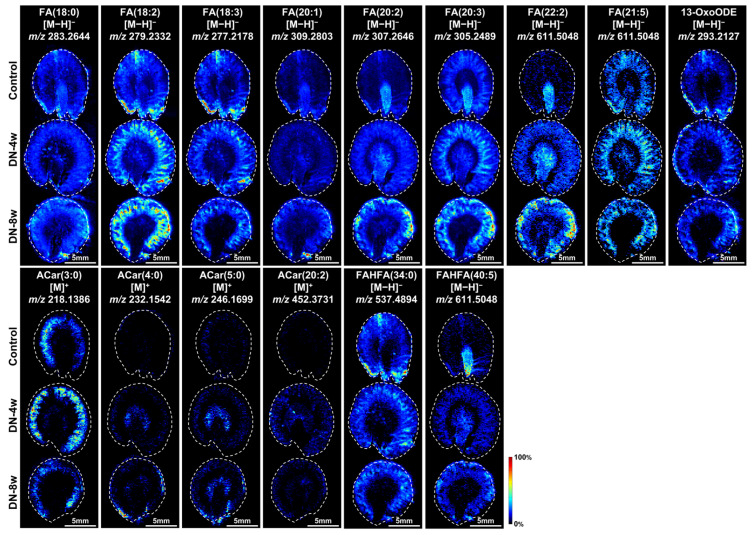
Spatial distribution of differentially abundant fatty acid subcategories in alloxan-induced diabetic rats. Acetyl carnitine, fatty acid esters of hydroxy fatty acids and free fatty acids detected through the AFADESI-MSI analysis in the kidneys of the groups. OC: outer cortex. JC: juxtamedullary cortex. OM: outer medulla. IM: inner medulla.

AFADESI–MSI revealed significant dysregulation of several free fatty acids (FFAs) were altered, including FA(18:0), FA(18:1), FA(22:6), and the oxidized lipid 13-OxoODE (Figure 8). Long-chain FFAs can drive renal lipotoxicity through ER stress, mitochondrial injury, and pro-inflammatory signaling cascades [32]. Moreover, oxidized lipid species such as 13-OxoODE can act as bioactive mediators that modulate inflammation and oxidative damage [33]. The increased abundance of oxidized FFAs is consistent with enhanced lipid peroxidation, which can exacerbate both glomerular and tubular injury during DN progression.

In parallel, multiple short- and medium-chain acylcarnitines, including propionylcarnitine, butyrylcarnitine, pivaloylcarnitine, and linoleylcarnitine (Figure 8). Because acylcarnitines are formed during mitochondrial fatty acid import and β-oxidation, their accumulation is commonly interpreted as a readout of incomplete fatty acid oxidation and mitochondrial substrate overload. Such mitochondrial stress can promote lipotoxicity, increase ROS production, and amplify inflammatory signaling, all of which contribute to DN pathogenesis [34]. Consistent with this mechanism, disturbed β-oxidation and acylcarnitine buildup have been linked to tubular epithelial injury and podocyte apoptosis, key cellular events underlying progressive renal dysfunction [35,36]. Collectively, these patterns suggest a shift away from efficient oxidative energy production toward maladaptive lipid handling in diabetic kidneys.

Notably, we also detected fatty acid esters of hydroxy fatty acids (FAHFAs), including FAHFA(34:0) and FAHFA(40:5) (support 8). FAHFAs have been proposed as endogenous lipid mediators involved in regulating inflammation and insulin sensitivity [37]. Their dysregulation in diabetic kidneys may therefore reflect disrupted lipid signaling and a weakened anti-inflammatory lipid milieu, further predisposing renal tissue to metabolic inflammation and injury.

### 4.4. Lipids Metabolism

**Figure 9:**
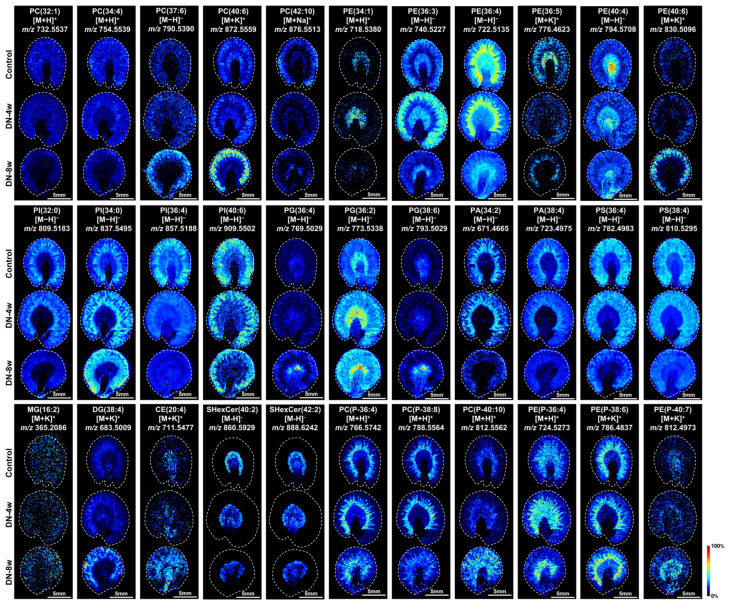
Spatial distribution of differentially abundant glyceryl ester subcategories in alloxan-induced diabetic rats. Phosphorylcholines and phosphatidylethanolamines, phosphatidylinositol, phosphatidylglycerol, phosphatidylserine and phosphatidic acid; monoacylglycerols (MGs) and diacylglycerols (DGs), and plasmalogens detected through the AFADESI-MSI analysis in the kidneys of the groups. OC: outer cortex. JC: juxtamedullary cortex. OM: outer medulla. IM: inner medulla.

AFADESI–MSI showed broad remodeling of renal lipid species, with significant changes in multiple glycerophospholipid classes, including phosphatidylethanolamines (PEs), phosphatidylcholines (PCs), phosphatidylglycerols (PGs), phosphatidylserines (PSs), and plasmalogens (e.g., PE(P-) and PC(P-)) (Figure 9). Because these lipids are core structural components of cellular membranes, their dysregulation implies altered membrane composition, with downstream consequences for membrane fluidity, vesicle trafficking, and signal transduction [38]. Plasmalogens are a major subclass of ether phospholipids with well-described radical-scavenging capacity; thus, reduced plasmalogen abundance is consistent with impaired lipid-phase antioxidant buffering and heightened oxidative vulnerability in diabetic kidneys [39].

In addition to bulk alterations in phospholipid profiles, several lysophospholipids (e.g., LPE(22:6) and LPI(16:0)) were significantly altered. Lysophospholipids function as bioactive signaling molecules capable of activating inflammatory pathways and modulating glomerular cell behavior, and they have been implicated in processes such as mesangial activation/expansion [40]. Therefore, the observed lysophospholipid imbalance provides a plausible mechanistic link between lipid dysregulation and progressive structural injury.

We also observed increased levels of monoacylglycerols (MGs) and diacylglycerols (DGs), indicating altered lipid metabolism and storage. Importantly, DGs act as second messengers that activate protein kinase C (PKC) [41]. PKC activation is a canonical pathway in DN and promotes extracellular matrix deposition and other maladaptive remodeling responses [42], contributing to glomerular basement membrane thickening and increased filtration barrier permeability.

Finally, the altered abundance of sulfated hexosylceramides (SHexCer) points to disruption of sphingolipid metabolism, which is increasingly recognized as a key determinant of renal injury [43]. Sphingolipid accumulation can drive inflammation, oxidative stress, and apoptosis in renal cells, and dysregulated sphingolipid signaling is closely associated with glomerular damage and proteinuria in diabetic kidney disease [44]. Together, these lipidomic changes support a model in which diabetic kidneys undergo coordinated perturbations in membrane lipids and lipid-derived signaling pathways that promote inflammatory and fibrotic injury.

### 4.5. Other Metabolic Pathways

**Figure 10:**
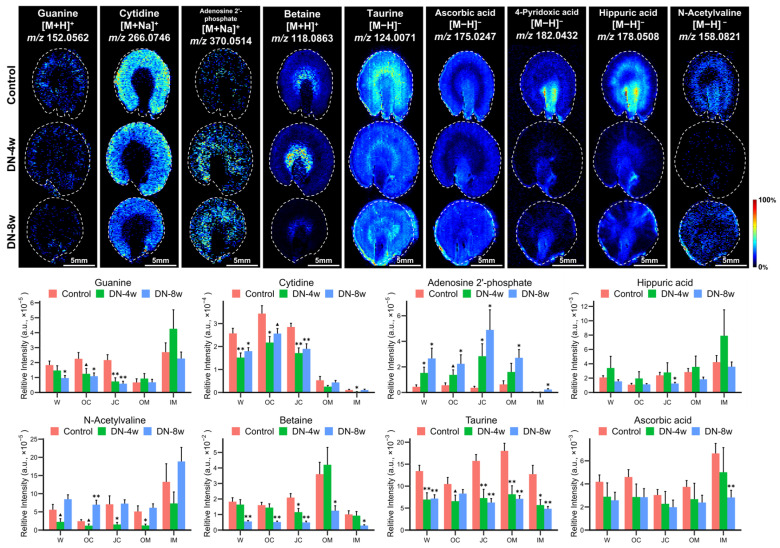
Spatial distribution of differentially abundant nucleotides and antioxidants uremic toxins in alloxan-induced diabetic rats with histogram of representative metabolites’ total ion current intensity per microdomain detected through the AFADESI-MSI analysis in the kidneys of the groups. OC: outer cortex. JC: juxtamedullary cortex. OM: outer medulla. IM: inner medulla. As compared to control group, ▴ *p* < 0.1, * *p* < 0.05 and ** *p* < 0.01 (*n* = 6; mean ± SEM).

Beyond amino acids and lipids, AFADESI–MSI captured additional metabolic changes implicating nucleotide metabolism, osmotic stress adaptation, antioxidant/cofactor depletion, and uremic toxin accumulation in diabetic kidneys (Figure 10). Alterations in cytidine and adenosine 2′-phosphate suggest enhanced nucleic acid turnover, which may reflect increased DNA/RNA damage and repair under oxidative stress [45]. Because purinergic signaling mediated by nucleotides and nucleosides modulates renal hemodynamics and immune activity, disruption of purine/pyrimidine-related metabolites may further reinforce inflammatory signaling and fibrotic remodeling during DN progression [46].

We also observed increased levels of betaine and taurine, consistent with a compensatory response to hyperglycemia-driven hyperosmolar stress. In addition to serving as compatible osmolytes, both metabolites can exert cytoprotective effects and contribute to antioxidant defenses, potentially buffering tubular cells against osmotic and oxidative injury [47]. In contrast, decreased ascorbic acid and pyridoxic acid (vitamin B6 metabolite) point to weakened antioxidant capacity and perturbed coenzyme homeostasis, which may favor ROS accumulation and aggravate microvascular and parenchymal injury in diabetic kidneys [48].

Finally, several uremic toxins and gut-derived metabolites, including hippuric acid and N-acetylarylamine, were elevated. These compounds are produced or influenced by intestinal microbial metabolism and are normally cleared by renal excretion; therefore, their accumulation is consistent with impaired renal clearance and/or increased gut-derived load. Elevated uremic solutes can further promote oxidative stress, stimulate inflammatory cytokine production, and accelerate fibrotic pathways, creating a feed-forward loop that worsens renal injury [49]. Together, these data highlight a potential contribution of the gut–kidney axis to metabolic dysregulation in alloxan-induced DN.

In summary, the spatial metabolomic alterations observed in alloxan-induced diabetic rat kidneys delineate a coherent metabolic cascade underlying diabetic nephropathy, characterized by coordinated disturbances in glucose utilization, mitochondrial energy metabolism, lipid handling, redox balance, and inflammatory signaling. Persistent hyperglycemia drives excessive polyol pathway flux and mitochondrial dysfunction, leading to energy deficiency and oxidative stress. These changes, in turn, promote incomplete fatty acid β-oxidation and lipid accumulation, as evidenced by elevated acylcarnitines and diacylglycerols, thereby exacerbating renal lipotoxicity and activating pathogenic signaling pathways such as protein kinase C. Concomitant remodeling of glycerophospholipids, sphingolipids, and ether lipids reflects compromised membrane integrity and altered lipid-mediated signaling, contributing to tubular and glomerular injury. Disruptions in amino acid and nucleotide metabolism further indicate enhanced oxidative damage, inflammatory activation, and fibrotic progression, while the accumulation of uremic toxins highlights impaired renal clearance and gut–kidney axis involvement. Complementary proteomic analysis at the early disease stage further supports these findings, with differentially expressed proteins enriched in pathways related to mitochondrial function, fatty acid metabolism, and oxidative stress. The concordance between protein-level changes and spatially resolved metabolite alterations reinforces the involvement of impaired bioenergetics and lipid dysregulation in DN, providing additional molecular context for the observed metabolic remodeling.

Notably, distinct differences in metabolite profiles were observed between the DN-4w and DN-8w groups. Renal glucose levels were higher in the DN-4w group but lower in the DN-8w group, indicating variation in glucose-related metabolic status between the two time points. Metabolites associated with glucose utilization and energy metabolism—such as glycerol-3-phosphate, sorbitol, acylcarnitines, and free fatty acids (e.g., FA(18:0), FA(18:2), and FA(18:3))—also exhibited differing patterns between groups, suggesting heterogeneous metabolic responses under diabetic conditions. In contrast, several metabolites, including creatine, histidine, arginine, cystine, threonine, and PI(36:4), showed consistent directional changes between DN-4w and DN-8w groups. These patterns may reflect sustained alterations in redox balance, inflammatory signaling, and membrane lipid metabolism in diabetic kidneys.

Taken together, these observations highlight distinct patterns of metabolic variation between the DN-4w and DN-8w groups. Some metabolites associated with glucose utilization exhibit variable changes between groups, whereas others related to oxidative stress, lipid metabolism, and cellular signaling show more consistent alterations. These findings suggest that multiple metabolic processes are differentially affected in diabetic kidneys and may reflect heterogeneous responses across biochemical pathways. The spatial metabolomics approach employed in this study provides a useful framework for characterizing such region-specific metabolic differences under diabetic conditions.

These findings are consistent with our previous AFADESI-MSI-based spatial metabolomics studies in HFD/STZ-induced type 2 diabetic rats and db/db mice, which revealed pronounced regional heterogeneity in amino acid, lipid, and energy metabolism pathways, particularly at advanced disease stages [5,6]. However, those models primarily represent insulin resistance-associated (type 2) diabetic nephropathy and therefore do not fully capture the metabolic features of type 1 diabetes-related renal injury. In this context, the present study extends our prior work by focusing on an alloxan-induced model of type 1 diabetes, providing new insight into the spatial metabolic landscape of DN in the absence of insulin resistance.

Consistent with earlier observations, we again identified disturbances in glucose utilization, lipid metabolism, and mitochondrial energy pathways. Importantly, the spatially resolved approach revealed that these alterations are not uniformly distributed but instead exhibit distinct compartment-specific patterns, highlighting differential vulnerability across renal regions such as the cortex and outer medulla. In addition, by examining multiple time points, this study provides further insight into stage-associated metabolic changes, suggesting a transition from early substrate-driven perturbations to later metabolic dysfunction. Together, these findings complement and extend previous work by demonstrating that, while core metabolic pathways are commonly affected across DN models, their spatial organization and temporal dynamics may differ between type 1 and type 2 diabetes-associated kidney injury.

This study has several limitations. The absence of an age-matched control group at the later time point restricts the ability to fully separate disease progression from age-related metabolic variation. In addition, a subset of metabolite identifications relied primarily on accurate mass matching and database annotation, which may introduce uncertainty, particularly for isomeric or low-abundance compounds. Furthermore, the lack of enzymatic or biochemical validation (e.g., enzyme activity assays or immunostaining) limits direct confirmation of the proposed metabolic pathway alterations. Despite these limitations, the spatial metabolomics approach used in this study provides consistent and biologically relevant insights into region-specific metabolic alterations in type 1 diabetes-associated diabetic nephropathy.

## 5. Conclusions

In this study, AFADESI-MSI-based spatial metabolomics was applied to characterize metabolic alterations in the kidneys of an alloxan-induced rat model of type 1 diabetes-associated diabetic nephropathy. The results demonstrate alterations in metabolites related to glucose metabolism, energy pathways, lipid metabolism, membrane lipids, and redox-related processes, with distinct spatial patterns across renal compartments. These findings highlight heterogeneous metabolic distributions among different renal microregions under diabetic conditions. Complementary proteomic data at the early stage provide additional context for the metabolic changes observed.

By focusing on a type 1 diabetes model, this work complements previous studies in type 2 diabetic models and provides new insight into the spatial organization and stage-associated features of metabolic perturbations in diabetic nephropathy. Overall, this study demonstrates the value of spatial metabolomics, with proteomic support, for resolving compartment-specific metabolic alterations and advancing the understanding of metabolic heterogeneity in diabetic nephropathy. These findings further highlight the potential of spatial metabolomics for identifying region-specific biomarkers and therapeutic targets in diabetic kidney disease.

## Data Availability

The original contributions presented in this study are included in the article/Supplementary Materials. Further inquiries can be directed to the corresponding author.

## Supplementary Materials

The following supporting information can be downloaded at: https://www.mdpi.com/article/10.3390/metabo16060355/s1. Supplementary Methods; Table S1. AFADESI–MSI analysis parameters of metabolites in cross-sectioned kidney tissue; Table S2. Liquid chromatography elution gradient of proteomics; Figure S1. Histogram of fold changes of significantly different metabolites across groups; Figure S2. Clustered heat map based on differentially abundant metabolites among Control group and alloxan-induced diabetic rats (DN-4w and DN-8w); Figure S3. KEGG enrichment of differential expression metabolites; Table S3. Cumulative R2X, cumulative R2Y, cumulative Q2 and Q2 intercepts after 200 permutations of each OPLS-DA analysis of each ROI in AFADESI-MSI; Figure S4. Histogram of representative metabolites total ion current intensity per microdomain involved in fatty acids metabolism detected through the AFADESI-MSI analysis in the kidneys of the groups; Figure S5. Histogram of representative metabolites total ion current intensity per microdomain of glycerol lipids detected through the AFADESI-MSI analysis in the kidneys of the groups; Figure S6. Histogram of representative metabolites total ion current intensity per microdomain of glycerophosphocholine and lysophospholipids and detected through the AFADESI-MSI analysis in the kidneys of the groups; Table S4. Differentially abundant metabolites obtained by positive AFADESI-MSI analysis of renal sections from control and alloxan diabetic model groups; Table S5. Differentially abundant metabolites obtained by negative AFADESI-MSI analysis of renal sections from control and alloxan diabetic model groups.
