# Supplementary material for: Mapping Spatiotemporal Metabolic Perturbations in Alloxan-Induced Diabetic Rat Kidneys Using Spatial Metabolomics and Proteomic Integration
Source: Metabolites. 2026 May 25;16(6):355. doi: 10.3390/metabo16060355 (PMC13304243; doi:10.3390/metabo16060355)
Supplement: Supplementary file 1 [file metabolites-16-00355-s001.zip › metabolites-4273799-supplementary.pdf]

Supporting Information

Mapping spatiotemporal metabolic perturbations in alloxan-induced diabetic rat kidneys by spatial metabolomics and proteomic integration

Tianfang Lan [1], Caiying Liu [1], Xingyu Zhang [1], Xiaoyu Zhang [1], Yuchen Liu[1], Wenxuan Shao[1], Zhonghua Wang [1, 2, *]

[1] *Center for Imaging and Systems Biology, College of Life and Environmental Sciences, Minzu University of China, Beijing, 100081, China*

[2] *Key Laboratory of Mass Spectrometry Imaging and Metabolomics (Minzu University of China), National Ethnic Affairs Commission, Beijing, 100081, China*

*Corresponding author:

Tel: +86 10-68933506;

Fax: +86 10-68933506;

E-mail: wangzhonghua@muc.edu.cn

Supplementary Methods

1. LC–MS/MS analysis of metabolites in homogenized kidney tissue

1.1 LC-MS/MS data acquisition

LC–MS/MS analyses were performed in both positive and negative ion modes using a Q-OT-qIT hybrid mass spectrometer (Orbitrap Fusion Lumos, Thermo Fisher Scientific, USA). Approximately 0.1 g of renal tissue was placed in a 2 mL centrifuge tube and homogenized with 0.4 mL of MeOH:H_2O (8:2, *v/v*). The homogenate was processed using a tissue grinder for 1 min, followed by centrifugation at 12,000 rpm for 10 min at 4 °C. The supernatant was collected and dried in a vacuum concentrator (~3 h), then reconstituted in 200 μL of H_2O:ACN (99:1, *v/v*) and vortexed for 5 min. After centrifugation at 12,000 rpm for 10 min at 4 °C, 120 μL of the supernatant was transferred to an LC autosampler vial for analysis. For targeted ions, MS and MS/MS data were acquired in full scan and $ddMS^2$ modes with normalized HCD collision energies of 15%, 30%, and 45%. MS/MS acquisition was performed at a resolution of 15,000, with a maximum injection time of 100 ms and a mass range of *m/z* 67–1000. Chromatographic separation was achieved on a reversed-phase Waters ACQUITY UPLC HSS T3 column (1.8 μm, 2.1 × 100 mm). The mobile phases consisted of 0.1% formic acid in water (A) and acetonitrile (B). The gradient program was as follows: 1–30% B (0–5 min), 30–65% B (5–8 min), 65% B (8–14 min), 65–99% B (14–18 min), 99% B (18–22 min), 99–1% B (22–23 min), and 1% B (23–30 min).

1.2 LC-MS/MS data analysis

LC–MS/MS raw data (.raw files) acquired from rat kidney tissue homogenates were converted to .mzXML format using MSConvert (ProteoWizard). Data processing was performed using the R-based XCMS package, including retention time alignment, peak detection, peak filtering, and feature matching, resulting in a data matrix containing m/z, retention time, and peak intensity information. Metabolite annotation was conducted by matching detected features against the Human Metabolome Database (HMDB, https://hmdb.ca/) based on accurate mass. Corresponding MS/MS spectra of annotated features were extracted using Xcalibur (Thermo Fisher Scientific) and further compared with reference spectra from public databases, including MassBank and PubChem, to support metabolite identification.

2. Proteomic analysis

2.1 Total Protein Extraction

Approximately 100 mg of kidney tissue from Control and DN-4w groups (n = 3) was individually ground in liquid nitrogen and lysed in SDT buffer (containing 100 mM NaCl) supplemented with 1/100 volume of dithiothreitol (DTT), followed by ultrasonication on ice for 5 min. The lysates were incubated at 95 °C for 8–15 min, cooled on ice for 2 min, and centrifuged at 12,000 × g for 15 min at 4 °C. The supernatant was collected and alkylated with iodoacetamide at room temperature in the dark for 1 h. Proteins were then precipitated by adding four volumes of pre-cooled acetone, followed by vortexing and incubation at −20 °C for at least 2 h. The samples were centrifuged at 12,000 × g for 15 min at 4 °C, and the resulting pellet was washed

with 1 mL of cold acetone. Finally, the protein pellet was fully dissolved in dissolution buffer (DB buffer) for subsequent analysis.

2.2 Trypsin treatment

Protein samples were adjusted to a final volume of 100 μL using DB lysis buffer (8 M urea, 100 mM TEAB, pH 8.5). Sequencing-grade trypsin and 100 mM TEAB buffer were added, and the mixture was incubated at 37 °C for 4 h. Subsequently, additional trypsin and $CaCl_2$ were added, and digestion was continued overnight at 37 °C. The reaction was terminated by adding formic acid to adjust the pH to < 3, followed by centrifugation at 12,000 × g for 5 min at room temperature. The supernatant was loaded onto a C18 desalting column, washed three times with washing buffer (0.1% formic acid, 3% acetonitrile), and eluted with elution buffer (0.1% formic acid, 70% acetonitrile). The eluates were collected and lyophilized for subsequent analysis.

2.3 LC-MS/MS Analysis (DIA mode)

Mobile phases were prepared as follows: solvent A, water containing 0.1% formic acid; solvent B, acetonitrile containing 0.1% formic acid. Lyophilized peptide samples were reconstituted in 10 μL of solvent A, centrifuged at 14,000 × g for 20 min at 4 °C, and approximately 200 ng of peptides was injected for LC–MS/MS analysis. Chromatographic separation was performed using a Vanquish Neo UHPLC system (Thermo Fisher Scientific) equipped with a C18 trap column (5 mm × 300 μm, 5 μm) and a C18 analytical column (PepMap™ Neo UHPLC, 150 μm × 15 cm, 2 μm), maintained at 50 °C. The gradient elution conditions are provided in Table S2. Mass spectrometric analysis was carried out on an Orbitrap Astral mass spectrometer (Thermo Fisher Scientific) using an Easy-Spray electrospray ionization (ESI) source. The spray voltage was set to 1.9 kV, and the ion transfer tube temperature was 290 °C. Data were acquired in data-independent acquisition (DIA) mode with a full MS scan range of m/z 380–980 at a resolution of 240,000 (at *m/z* 200). The automatic gain control (AGC) target was set to 500%, with a precursor isolation window of 2 Th and 300 DIA windows. The normalized collision energy (NCE) was set to 25%. MS/MS spectra were acquired over an m/z range of 150–2000 with a resolution of 80,000 (Astral) and a maximum injection time of 3 ms. Raw data were recorded in .raw format for subsequent analysis.

2.4 Protein Identification and Quantitation

Raw LC–MS/MS data were processed using DIA-NN (version 1.8.1) for protein identification and quantification against the UniProt Rattus norvegicus database. The search parameters included a precursor mass tolerance of 10 ppm and a fragment ion tolerance of 0.02 Da. Carbamidomethylation of cysteine was set as a fixed modification, while oxidation of methionine and protein N-terminal acetylation were considered as variable modifications. Up to one missed cleavage site was allowed. To ensure high-confidence identifications, peptide-spectrum matches (PSMs) and proteins were filtered at a false discovery rate (FDR) of <1% at both the peptide and protein levels. Only high-confidence peptides and proteins were retained for further analysis. Differentially expressed proteins (DEPs) were defined based on fold change (FC) thresholds and statistical significance criteria, as described in the corresponding section.

Table S1 AFADESI-MSI parameters for kidney tissue metabolite imaging

**Parameters**	**Values**
Ion Source Votage (V)	±7000
Ion Transfer Tube Temp (°C)	350
Nebulizing Gas Pressure (MPa)	0.6
Evacuation Rate (L/min)	45
Spraying Angle	60°
Sparying Solvent	ACN/H_2O (8:2, *v/v*)
Sparying Flow Rate (μL/min)	7
X Axis Scanning Rate (mm/s)	0.2
Y Axis Stepping (mm)	0.2
Distance from Nebulizer to Sample (mm)	0.6
Distance from Neublizer to Ion Transfer Tube (mm)	3
Distance from Ion Transfer Tube to Skimmer (mm)	10
Scan Type	Full scan
Scan Range (*m/z*)	100-1000
Ion Transfer Tube Temp (°C)	350
Orbitrap Resolution	120000
Microscans	1
Maximum Injection Time (ms)	70
AGC Target (%)	100
RF Lens (%)	60

Table S2. Liquid chromatography gradient conditions for proteomic analysis

Time	flow rate (nL/min)	mobile phase A (%)	mobile phase B（%）
0	2.5	96	4
0.2	1.3	96	4
0.3	0.8	92	8
0.5	0.8	92	8
14.2	0.8	77.5	22.5
21.1	0.8	65	35
21.5	2.5	45	35
21.5			
21.9	2.5	1	99
22.6	2.5	1	99
22.6			

Supplementary Figures

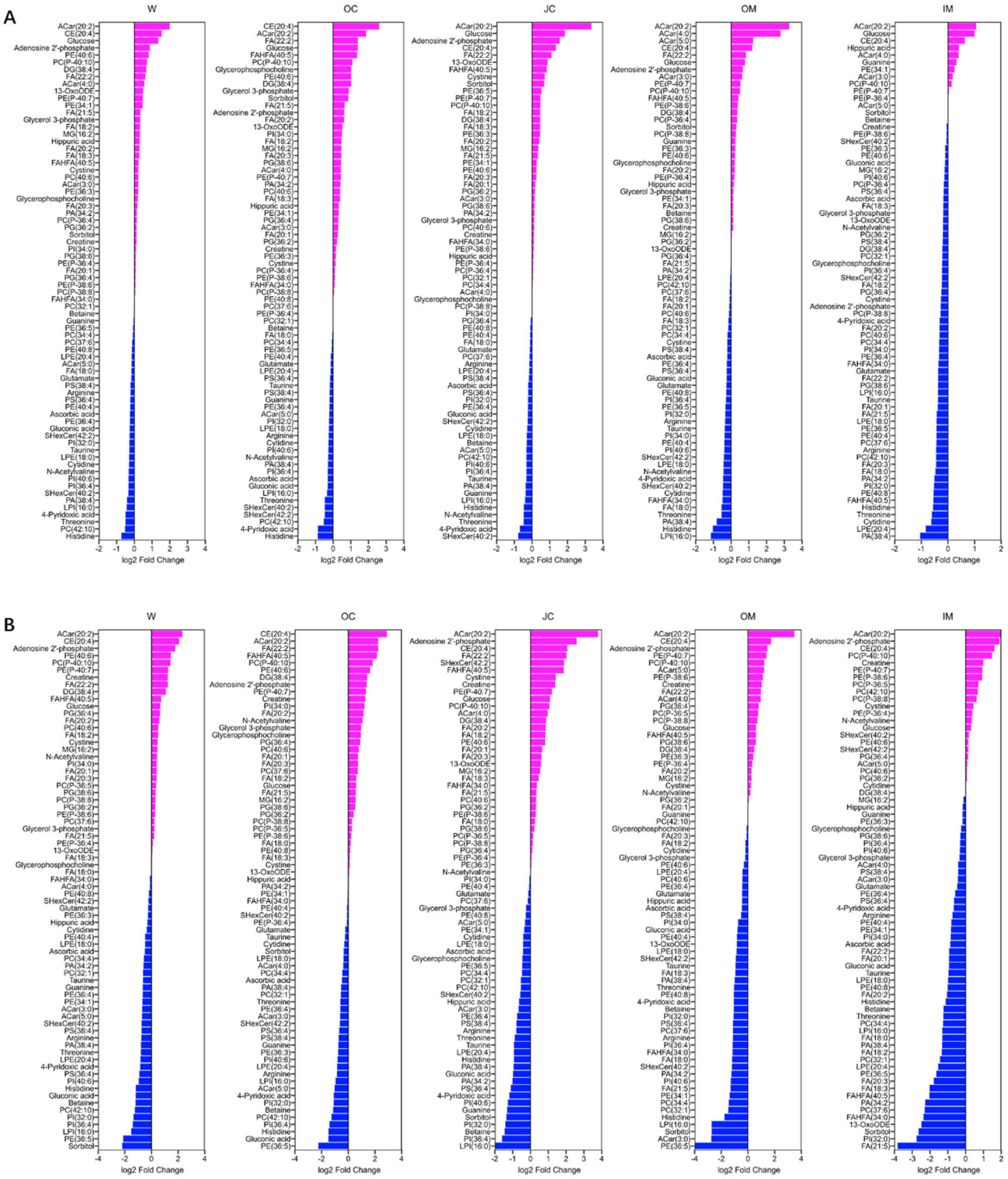

Fig. S1. Histogram of fold changes for significantly altered metabolites across groups. (A) DN-4w vs. Control. (B) DN-8w vs. Control.

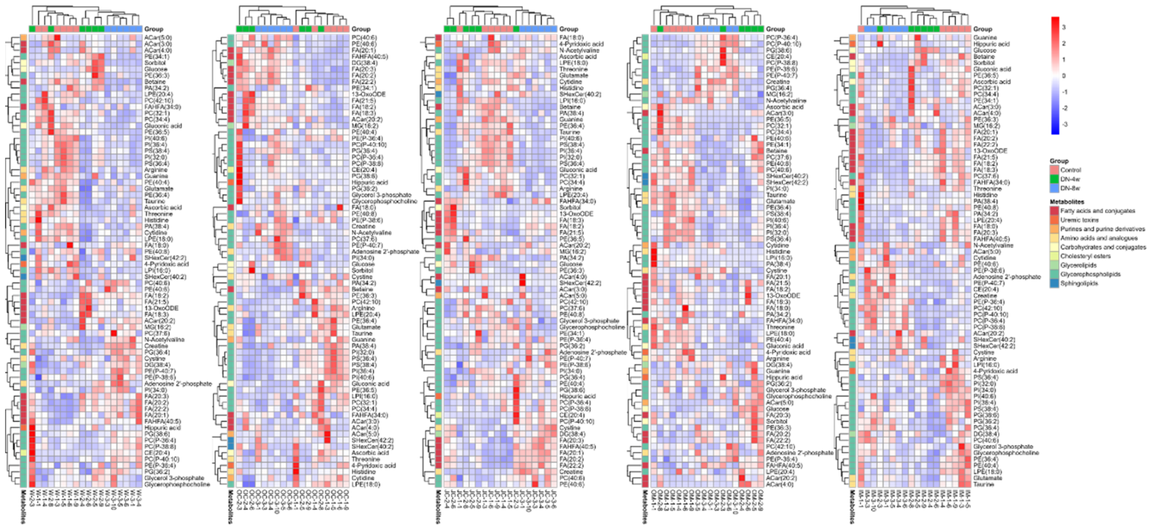

Fig. S2. Hierarchical clustering heatmap of differentially abundant metabolites across Control and alloxan-induced diabetic groups (DN-4w and DN-8w). W: Whole kidney; OC: Outer cortex; JC: Juxtamedullary cortex; OM: Outer medulla; IM: Inner medulla.

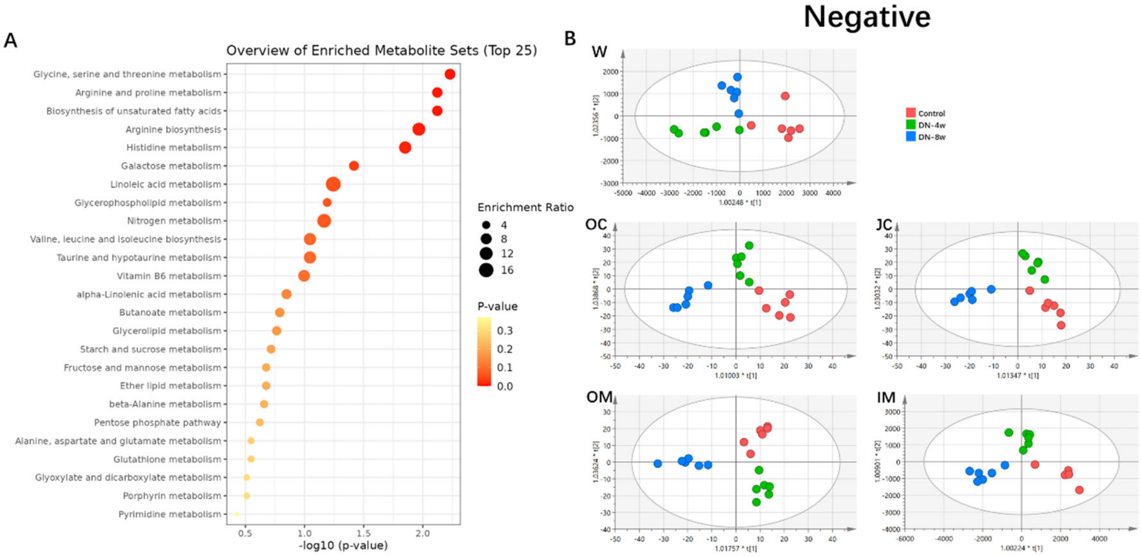

Fig S3. KEGG pathway enrichment analysis of differentially abundant metabolites and OPLS-DA score plots. OPLS-DA models were constructed based on positive and negative ion mode AFADESI-MSI data from the Control (n = 6), DN-4w (n = 6), and DN-8w (n = 6) groups. W: Whole kidney; OC: Outer cortex; JC: Juxtamedullary cortex; OM: Outer medulla; IM: Inner medulla.

Table S3. Summary of OPLS-DA model validation parameters for each ROI based on AFADESI-MSI data, including cumulative R^2X, R^2Y, Q^2, and Q^2 intercepts from 200 permutation tests.

ROI	Polarity	Cum. R^2X	Cum. R^2Y	Cum. Q^2	Q^2 intercept
IM	Positive	0.669	0.860	0.239	-0.538
OM	Positive	0.768	0.908	0.560	-0.594
JC	Positive	0.707	0.938	0.504	-0.597
OC	Positive	0.723	0.932	0.614	-0.814
W	Positive	0.922	0.999	0.792	-0.521
IM	Negative	0.606	0.900	0.305	-0.035
OM	Negative	0.543	0.821	0.222	-0.021
JC	Negative	0.590	0.784	0.209	-0.065
OC	Negative	0.564	0.775	0.252	-0.130
W	Negative	0.657	0.807	0.299	-0.211

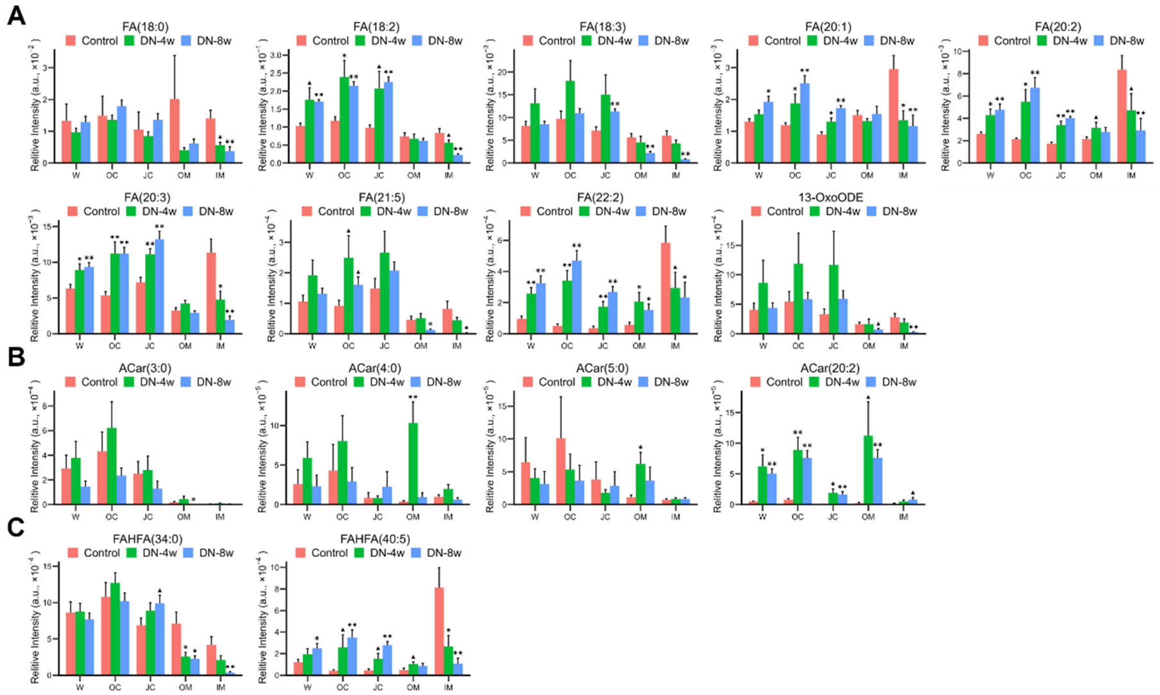

Fig. S4. Bar plots of TIC-normalized intensities of representative fatty acid–related metabolites across renal microdomains in different groups, as detected by AFADESI-MSI.OC: Outer cortex; JC: Juxtamedullary cortex; OM: Outer medulla; IM: Inner medulla. ▲ $P < 0.1$, * $P < 0.05$, ** $P < 0.01$ (n=6; mean±SEM)

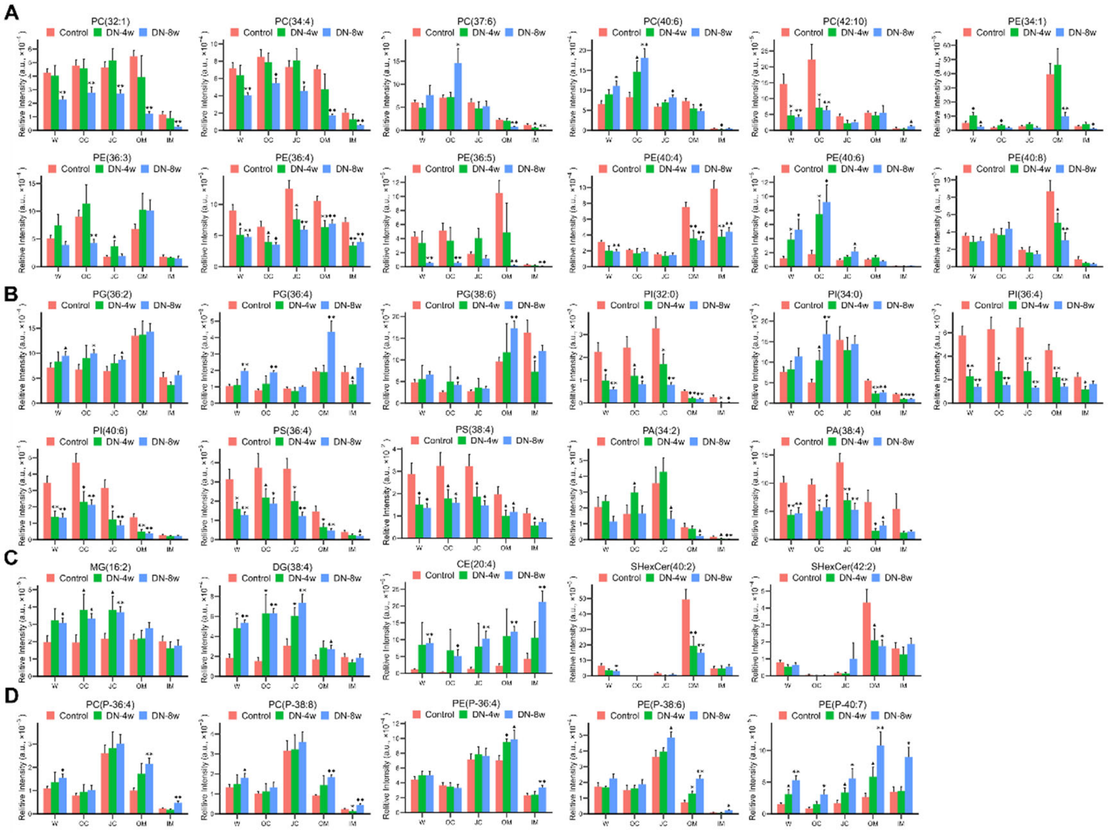

Fig. S5. Bar plots of TIC-normalized intensities of representative glycerolipid-related metabolites across renal microdomains in different groups, as detected by AFADESI-MSI.(A) Phosphatidylcholine (PC) and phosphatidylethanolamine (PE); (B) Other phospholipids; (C) Acetal glycerol lipids and sphingolipids; (D) Plasmalogens. OC: Outer cortex; JC: Juxtamedullary cortex; OM: Outer medulla; IM: Inner medulla. ▲ $P < 0.1$, * $P < 0.05$, ** $P < 0.01$ (n=6; mean±SEM).

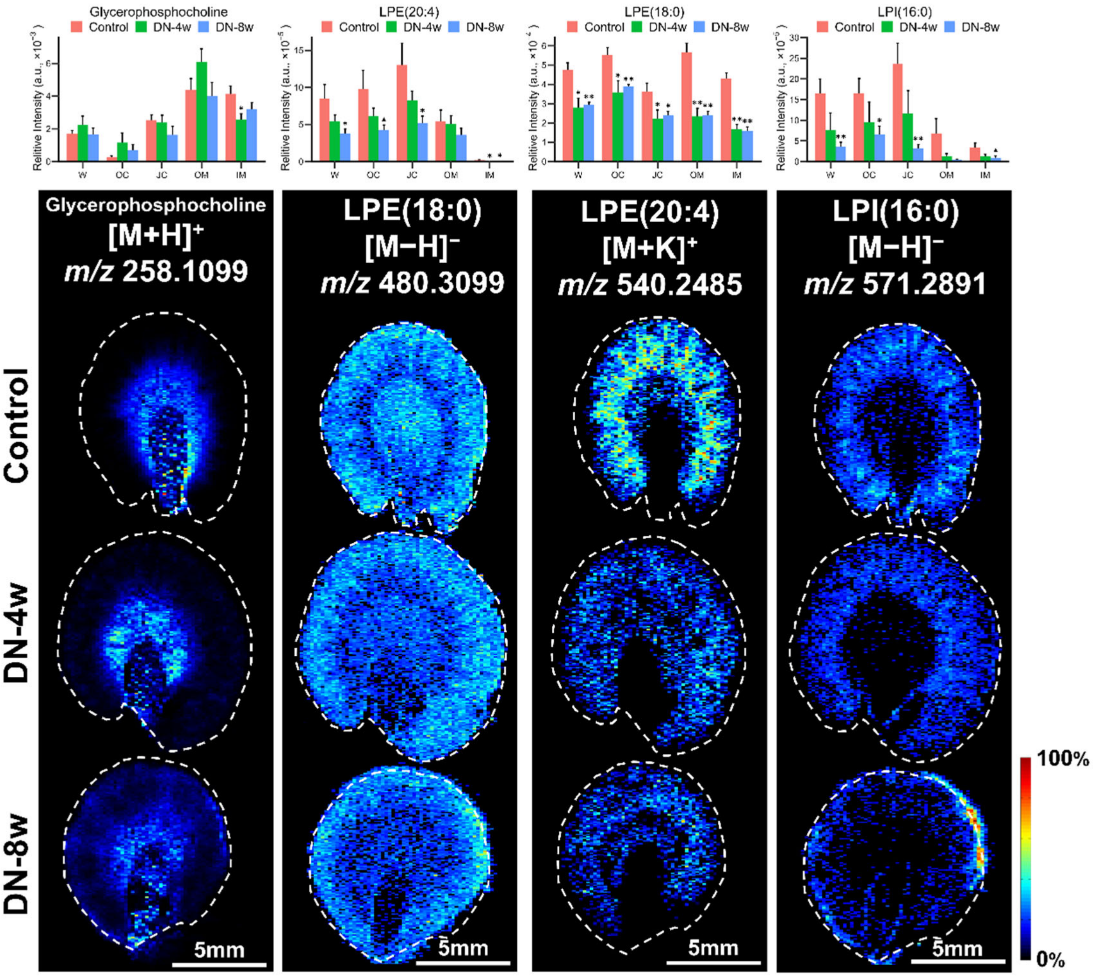

Fig. S6. Bar plots of TIC-normalized intensities and AFADESI-MSI ion images of representative glycerophosphocholine and lysophospholipid metabolites across renal microdomains in different groups. OC: Outer cortex; JC: Juxtamedullary cortex; OM: Outer medulla. ▲ $P < 0.1$, * $P < 0.05$, ** $P < 0.01$ (n=6; mean±SEM).

Table S4 Differentially abundant metabolites identified by positive-ion mode AFADESI-MSI in renal tissue sections from Control and alloxan-induced diabetic groups.

Metabolite Identification	Molecular Formula	Adduct ion	Relative Error(ppm)	Measured *m/z*	MS/MS
Betaine	C5H11NO2	$[M+H]^+$	−0.31	118.0863	55.0543, 58.0652, 59.0730, 72.0907
Guanine	C5H5N5O	$[M+H]^+$	−3.04	152.0562	
Creatine	C4H9N3O2	$[M+K]^+$	0.41	170.0327	90.0545, 132.0762
Histidine	C6H9N3O2	$[M+Na]^+$	−0.33	178.0586	
Arginine	C7H16N4O2	$[M+H]^+$	0.01	189.1346	158.0922, 130.0860, 116.0730, 70.0651, 60.0808
Glycerol 3-phosphate	C3H9O6P	$[M+Na]^+$	−0.11	195.0029	
Glucose	C6H12O6	$[M+Na]^+$	0.23	203.0526	
Propionylcarnitine	C10H19NO4	$[M+H]^+$	−0.49	218.1386	
Butyrylcarnitine	C11H21NO4	$[M+H]^+$	−0.25	232.1542	60.0805, 85.0280, 144.1015, 173.0805
Pivaloylcarnitine	C12H23NO4	$[M+H]^+$	−0.45	246.1699	
Glycerophosphocholine	C8H20NO6P	$[M+H]^+$	−0.60	258.1099	60.0810,86.0966,104.1071, 184.0734
Cytidine	C9H13N3O5	$[M+Na]^+$	−0.31	266.0746	
Cystine	C6H12N2O4S2	$[M+K]^+$	−0.47	278.9869	
MAG(16:2)	C19H34O4	$[M+K]^+$	−0.61	365.2086	
Adenosine 2'-phosphate	C10H14N5O7P	$[M+Na]^+$	−2.34	370.0514	
ACar(20:2)	C27H50NO4+	$[M]^+$	−0.79	452.3731	
LPE(20:4)	C25H44NO7P	$[M+K]^+$	−0.37	540.2485	
DG(38:4)	C41H72O5	$[M+K]^+$	−0.39	683.5009	
CE(20:4)	C47H76O2	$[M+K]^+$	−0.03	711.5477	
PE(34:1)	C39H76NO8P	$[M+H]^+$	−0.08	718.5380	
PE(P-36:4)	C41H74NO7P	$[M+H]^+$	−0.38	724.5273	
PC(32:1)	C40H78NO8P	$[M+H]^+$	−0.13	732.5537	
PC(34:4)	C42H76NO8P	$[M+H]^+$	−2.95	754.5359	
PC(P-36:5)	C44H80NO7P	$[M+H]^+$	−0.47	766.5742	
PE(36:5)	C41H72NO8P	$[M+K]^+$	−0.50	776.4623	
PE(P-38:6)	C43H74NO7P	$[M+K]^+$	0.29	786.4837	
PC(P-38:8)	C46H78NO7P	$[M+H]^+$	−3.16	788.5564	
PE(P-40:7)	C45H76NO7P	$[M+K]^+$	−2.23	812.4973	
PC(P-40:10)	C48H78NO7P	$[M+H]^+$	−3.30	812.5562	
PE(40:6)	C45H78NO8P	$[M+K]^+$	−0.05	830.5096	
PC(40:6)	C48H84NO8P	$[M+K]^+$	−0.86	872.5559	184.0729
PC(42:10)	C50H80NO8P	$[M+Na]^+$	−0.15	876.5513	104.1066, 124.9994, 184.0727

Table S5 Differentially abundant metabolites identified by negative-ion mode AFADESI-MSI in renal tissue sections from Control and alloxan-induced diabetic groups.

Metabolite Identification	Molecular Formula	Adduct ion	Relative Error(ppm)	Measured *m/z*	MS/MS
Threonine	C4H9NO3	$[M-H]^-$	−2.80	118.0507	72.0237
Taurine	C2H7NO3S	$[M-H]^-$	−2.79	124.0071	79.9571, 124.0072
Glutamate	C5H9NO4	$[M-H]^-$	−1.61	146.0457	102.0548, 128.0341
N-Acetylvaline	C7H13NO3	$[M-H]^-$	−1.04	158.0821	
Ascorbic acid	C6H8O6	$[M-H]^-$	−0.72	175.0247	
Hippuric acid	C9H9NO3	$[M-H]^-$	−1.02	178.0508	
Sorbitol	C6H14O6	$[M-H]^-$	−1.32	181.0716	
4-Pyridoxic acid	C8H9NO4	$[M-H]^-$	−0.69	182.0458	
Gluconic acid	C6H12O7	$[M-H]^-$	−0.21	195.0510	
FA(18:3)	C18H30O2	$[M-H]^-$	1.88	277.2178	59.0162, 259.2191
FA(18:2)	C18H32O2	$[M-H]^-$	0.89	279.2332	59.0136, 261.2235
FA(18:0)	C18H36O2	$[M-H]^-$	0.37	283.2644	59.0128, 265.2545
13-OxoODE	C18H30O3	$[M-H]^-$	1.86	293.2127	
FA(20:3)	C20H34O2	$[M-H]^-$	1.04	305.2489	
FA(20:2)	C20H36O2	$[M-H]^-$	1.12	307.2646	59.0133, 289.2475, 307.2633
FA(20:1)	C20H38O2	$[M-H]^-$	1.21	309.2803	104.9549, 309.2780
FA(21:5)	C21H32O2	$[M-H]^-$	1.13	315.2333	
FA(22:2)	C22H40O2	$[M-H]^-$	1.02	335.2959	59.0138, 317.2850, 335.296
LPE(18:0)	C23H48NO7P	$[M-H]^-$	0.61	480.3099	78.9594, 140.0111, 196.0376, 283.2647
FAHFA(34:0)	C34H66O4	$[M-H]^-$	1.12	537.4894	
LPI(16:0)	C25H49O12P	$[M-H]^-$	0.31	571.2891	
FAHFA(40:5)	C40H68O4	$[M-H]^-$	0.57	611.5048	
PA(34:2)	C37H69O8P	$[M-H]^-$	1.18	671.4665	
PA(38:4)	C41H73O8P	$[M-H]^-$	0.73	723.4975	
PE(36:3)	C41H76NO8P	$[M-H]^-$	−1.25	740.5227	
PG(36:4)	C42H75O10P	$[M-H]^-$	0.49	769.5029	279.2330
PG(36:2)	C42H79O10P	$[M-H]^-$	0.05	773.5338	
PS(36:4)	C42H74NO10P	$[M-H]^-$	0.59	782.4983	
PE(40:8)	C45H74NO8P	$[M-H]^-$	2.70	786.5100	
PG(38:6)	C44H75O10P	$[M-H]^-$	0.53	793.5029	
PE(40:4)	C45H82NO8P	$[M-H]^-$	0.42	794.5708	
PI(32:0)	C41H79O13P	$[M-H]^-$	−0.31	809.5183	
PS(38:4)	C44H78NO10P	$[M-H]^-$	0.44	810.5295	281.2454, 723.4972
PI(34:0)	C43H83O13P	$[M-H]^-$	−0.42	837.5495	
PI(36:4)	C45H79O13P	$[M-H]^-$	0.26	857.5188	
SHexCer(d40:2)	C46H87NO11S	$[M-H]^-$	0.20	860.5929	
SHexCer(d42:2)	C48H91NO11S	$[M-H]^-$	0.19	888.6241	
PI(40:6)	C49H83O13P	$[M-H]^-$	0.29	909.5502	
